# Predictive risk factors for early recurrence in patients with localized pancreatic ductal adenocarcinoma who underwent curative-intent resection after preoperative chemoradiotherapy

**DOI:** 10.1371/journal.pone.0264573

**Published:** 2022-04-04

**Authors:** Yasuhiro Murata, Toru Ogura, Aoi Hayasaki, Kazuyuki Gyoten, Takahiro Ito, Yusuke Iizawa, Takehiro Fujii, Akihiro Tanemura, Naohisa Kuriyama, Masashi Kishiwada, Hiroyuki Sakurai, Shugo Mizuno

**Affiliations:** 1 Department of Hepatobiliary Pancreatic and Transplant Surgery, Mie University Graduate School of Medicine, Tsu, Mie, Japan; 2 Clinical Research Support Center, Mie University Hospital, Tsu, Mie, Japan; Campus Bio Medico University, ITALY

## Abstract

**Background:**

The optimal surgical indication after preoperative chemoradiotherapy (CRT) remains a subject of debate for patients with pancreatic ductal adenocarcinoma (PDAC) because early recurrence often occurs even after curative-intent resection. The present study aimed to identify perioperative risk factors of early recurrence for patients with PDAC who underwent curative-intent resection after preoperative CRT.

**Methods:**

Two hundred three patients with PDAC who underwent curative-intent resection after preoperative CRT from February 2005 to December 2018 were retrospectively analyzed. The optimal threshold for differentiating between early and late recurrence was determined by the minimum *p*-value approach. Multivariate regression analysis was performed to identify predictive factors for early recurrence.

**Results:**

In 130 patients who developed recurrence after resection, 52 who had an initial recurrence within 12 months were defined as the early recurrence group, and the remaining 78 were defined as the late recurrence group. The incidence of hepatic recurrence was significantly higher in the early recurrence group than in the late recurrence group (39.7 vs. 15.4%). The early recurrence group had significantly lower 3-year rates of post-recurrence and overall survival than the late recurrence group (4.0 and 10.7% vs. 9.8 and 59.0%, respectively). Serum level of CA19-9 before surgery ≥56.8 U/ml was identified as an independent risk factor for early recurrence (OR:3.07, 95%CI:1.65–5.73, *p*<0.001) and associated with a significantly higher cumulative incidence rate of hepatic recurrence and lower rates of recurrence-free and overall survival.

**Conclusion:**

Serum level of CA19-9 before surgery after preoperative CRT was a strong predictive factor for early recurrence.

## Introduction

Pancreatic ductal adenocarcinoma (PDAC) is one of the most lethal of all major human malignancies. Although R0 resection remains the only chance for cure, many patients are considered borderline resectable or locally advanced unresectable at the time of initial diagnosis because the anatomical features of the pancreas are surrounded by major vessels [1]. Even in the most favorable cohorts with resectable PDAC, up to 80% of patients developed tumor recurrence after resection [2]. The preoperative CA19-9 levels more than 500 IU/mL and the existence of regional lymph node metastasis, which are referred as biological definition of borderline resectable PDAC, are expression of a more aggressive biological disease with a higher risk of early recurrence after surgery and a poor prognosis, even if the tumor is anatomically resectable [3]. Therefore, the current therapeutic approach for patients with PDAC has become increasingly multimodal and more patients are being treated with preoperative therapy. Preoperative chemoradiotherapy (CRT) offers potential theoretical benefits, including control of systemic micrometastasis and potential downstaging of the primary tumor with a consequent reduction in the risks of a microscopically positive resection (R1), resulting in an improved outcome [1, 4, 5].

The indication criteria for surgery after preoperative CRT has been the subject of considerable debate. Early recurrence of PDAC often occurs even in patients who underwent curative-intent resection after preoperative CRT, and the prognosis of patients with early recurrence is usually dismal. Therefore, it is important to identify clinical features in patients who develop early recurrence after resection for optimizing surgical indication after preoperative CRT.

Although the term “early recurrence” after surgery is often used in both academic and clinical settings in various cancers, a clear definition remains controversial for patients with PDAC. Early recurrence after surgery is recognized as a serious condition that has a poor prognosis in patients with initially resectable PDAC who underwent up-front surgery [6], and the 12-month period after pancreatectomy has been reported as the optimal threshold for differentiating between early and late recurrence in patients with PDAC undergoing up-front surgical resection [7, 8]. However, there is no consensus regarding the optimal threshold between early and late recurrence in patients undergoing resection after preoperative CRT, and the clinicopathological features of early recurrence remain unknown. The present study aimed to determine the optimal definition and to elucidate predictive risk factors for early recurrence after curative-intent resection for patients who received preoperative CRT.

## Materials and methods

### Study population

Among 339 patients with cytologically/histologically proven PDAC who were enrolled in the preoperative CRT protocol at Mie University Hospital from February 2005 to December 2018, 203 patients who underwent curative-intent resection after preoperative gemcitabine- or S-1 plus gemcitabine-based CRT were enrolled in the present study. Curative-intent resection was defined as the absence of apparent tumor residue in the surgical field, indicating R0 or R1 resection, without liver metastasis or macroscopic peritoneal dissemination. The exclusion criteria were a grossly positive resection margin (R2) and synchronous distant disease at the time of resection after preoperative CRT. A diagnosis of PDAC was confirmed by cytology or histology via endoscopic ultrasonography-guided fine-needle aspiration biopsy. Tumor resectability was determined by tetraphasic multidetector 64-row contrast-enhanced CT (MDCT) with thin slices at intervals of 1.00 mm according to a defined pancreatic protocol and was classified by the Japan Pancreas Society (JPS) classification [9]. The resectability of the 203 patients was classified into resectable (R) (n = 68), borderline resectable (BR) (n = 66), BR with portal vein contact (BR-PV) (n = 32), BR with arterial contact (BR-A) (n = 34), and unresectable locally advanced (UR-LA) (n = 69) at the initial visit. Patients were excluded when they showed distant metastatic lesions at the time of diagnosis. Informed consent was obtained from all patients, and this study was approved by the ethics committee of Mie University Graduate School of Medicine (no. 118 and no. 3021). The clinical and follow-up information were obtained from a prospectively maintained database and verified by reviewing patient medical records.

### Treatment protocol of CRT and surgery

Among the 203 patients, 64 received gemcitabine-based CRT (G-CRT) from February 2005 to October 2011 [10], and 139 were administered S-1 plus gemcitabine-based CRT (GS-CRT) from November 2011 to December 2018, as previously reported [11]. As for GS-CRT, patients were administered S1 orally twice daily at a dose of 60 mg/m^2^ per day on days through 21 of a 28-day cycle and an infusion of gemcitabine at a dose of 600 mg/m^2^ on days 8, 22, 36 and 50. Patients were treated with concurrent three-dimensional conformal radiotherapy as previously reported [11]. The gross tumor volume (GTV), including the gross tumor volume (GTVp) and lymph nodes of more than 1cm in diameter, was defined based on the CT images. The clinical target volume (CTV) was defined as GTV with a 5 to 10-mm margin and included the neuroplexus region of celiac axis (CA) and/or superior mesenteric artery (SMA) when the GTVp was located within 10 mm from the tunica adventitia of CA and/or SMA. The planning target volume was defined as the CTV with internal margin plus a set-up margin. The total radiation dose was 45 to 50.4 Gy in 25 to 28 fractions. Upon completion of CRT, all patients underwent restaging evaluation by MDCT. Radiographic responses were determined by comparing pretreatment and post-CRT CT scans. Tumor size (the sum of the long and short axes of the tumor) and carbohydrate antigen (CA) 19–9 level before CRT were compared with those measured at the time of reassessment after CRT. For conditional patient factors, the neutrophil-to-lymphocyte ratio (NLR) [12] and the prognostic nutritional index (PNI) [13] during CRT were assessed as follows: NLR (neutrophils/lymphocytes in μL/μL) and PNI (10 × albumin [g/dL] + 0.005 × total lymphocyte count [per mm^3^]).

The patients in which local down-staging (e.g., from UR-LA to BR) was achieved were planned to undergo surgery. Among the patients in whom tumor down-staging was not achieved, those with no encasement and deformity of major arteries such as the celiac axis, superior mesenteric artery (SMA), or jejunal arteries were offered surgery. Normalization or a remarkable decrease in serum CA19-9 level were criteria for surgical decision-making. Patients who were considered to have a contraindication of surgery continued receiving chemotherapy (gemcitabine for G-CRT or gemcitabine plus S-1 for GS-CRT) and underwent reevaluation by MDCT after two cycles of additional chemotherapy. Surgery was planned at the time when it was considered an indication by the reevaluation. The median duration of the preoperative treatment was 3.6 (1.5–17.2) months; 2.9 (1.5–8.4) months for G-CRT and 3.9 (2.7–17.2) months for GS-CRT.

At laparotomy, pancreatectomy was performed when there was no distant metastasis. Pancreaticoduodenectomy was performed with the nerve plexus hanging maneuver using the anterior approach, which helps to secure a negative surgical margin around the SMA [14]. Distal pancreatectomy was performed by radical antegrade modular pancreatosplenectomy [15]. Resection and reconstruction of the portal vein (PV) and/or superior mesenteric vein (SMV) were performed when the surgeon could not separate the pancreatic head from these vessels without leaving gross tumor on the vessel. When limited involvement of the common hepatic artery was identified, segmental resection and reconstruction were performed. Postoperative complications were graded according to the Clavien–Dindo classification [16].

### Postoperative treatment and follow-up

Within 6 weeks of surgery, we planned to initiate adjuvant chemotherapy with gemcitabine or S-1 and continue for at least 6 months, as previously reported [11]. All patients were evaluated as follows: physical examination every month, laboratory tests including serum levels of CA19-9 (normal 37 U/mL) every 2 or 3 months, and MDCT every 3 months within 2 years and thereafter every 6 months after surgery. However, when the serum levels of the tumor markers increased, the patients were evaluated by MDCT. Sites of recurrent disease were documented at the time of initial recurrence. Patients with recurrence and a good performance status were generally further treated with systemic chemotherapy. The day of the final follow-up was August 31, 2020, and no follow-up was missed. The median follow-up period was 28.5 (3.5–162.9) months for the subjects of the present study.

### Pathological assessment

The resected specimens were fixed in a formalin solution and embedded in paraffin blocks. A 3-μm section was obtained from each block and stained with hematoxylin and eosin. Pathological examination was performed by board-certified pathologists. The degree of residual tumor (R status) and histological response to preoperative CRT were evaluated according to the definition of JPS classification which adopted the 0mm clearance rule [9, 17]. R1 represented a microscopically incomplete resection with 0 mm clearance, and R0 indicated no residual tumor. According to the JPS classification system, the histological response to preoperative CRT was evaluated [9, 17]. The estimated rate (%) of residual tumor was defined as the volume of cancer cells considered viable / the estimated tumor volume before treatment. The grading of histological response is shown based on the estimated rate of residual tumor as follows: grade 1 (poor response): 50% or more (1a: 90% or more, 1b: 50% or more and less than 90%), grade 2 (moderate response): 10% or more and less than 50%, grade 3 (marked response): less than 10%, and grade 4 (complete response): no viable cancer cells are present.

### Statistical analysis

Continuous and categorical variables were summarized as the median (range) and frequency in each group and were compared between groups using the Mann–Whitney and chi-squared tests. The date of initial treatment was chosen as the starting point for the measurement of overall survival time after initial treatment. Patients who were alive were censored at the time of their last disease evaluation for analysis of overall survival (OS). Survival curves were constructed using the Kaplan–Meier method and compared between groups using the log-rank test.

Of the 203 patients, 130 patients (64.0%) experienced recurrence after preoperative CRT followed by curative-intent resection. The 130 patients were divided into two groups: early and late recurrence after curative-intent resection. A minimum *p-*value approach was used to evaluate the optimal threshold of the interval between curative-intent resection after CRT and recurrence to determine the optimal cut-off point with the lowest *p-*value to divide the patients into early and late recurrence cohorts based on the difference of post-recurrence survival (PRS) [7, 8]. Since follow-up CT was planned to perform every 3 months within 2 years after surgery in the present study, the cut-off point was determined to be the month with the lowest *p*-value among the months in multiples of 3 (months 3, 6, 12, 15, 18, and 21 after surgery). The clinicopathological data were analyzed and compared between the early and late recurrence groups.

Receiver operating characteristics (ROC) curves were constructed to estimate the optimal threshold for serum CA19-9 levels before surgery and blood loss during surgery as a perioperative risk factor for early recurrence. The optimal cut-off value was determined using ROC curve analysis. Associations between potential risk factors and early recurrence were assessed by univariate analysis, and the factors, of which *p*-value was determined to be less than 0.05 by univariate analysis, were entered into a multivariate regression analysis to ascertain independent predictive factors. Results were presented as odds ratios (ORs) with a corresponding 95% confidence interval (CI). *p*-values less than 0.05 were considered statistically significant. All statistical analyses were performed with JMP^®^ Pro software (version 13.0.0, SAS Institute Inc., Cary, NC, USA).

## Results

### Optimal cut-off threshold between early and late recurrence after surgery

**Fig 1:**
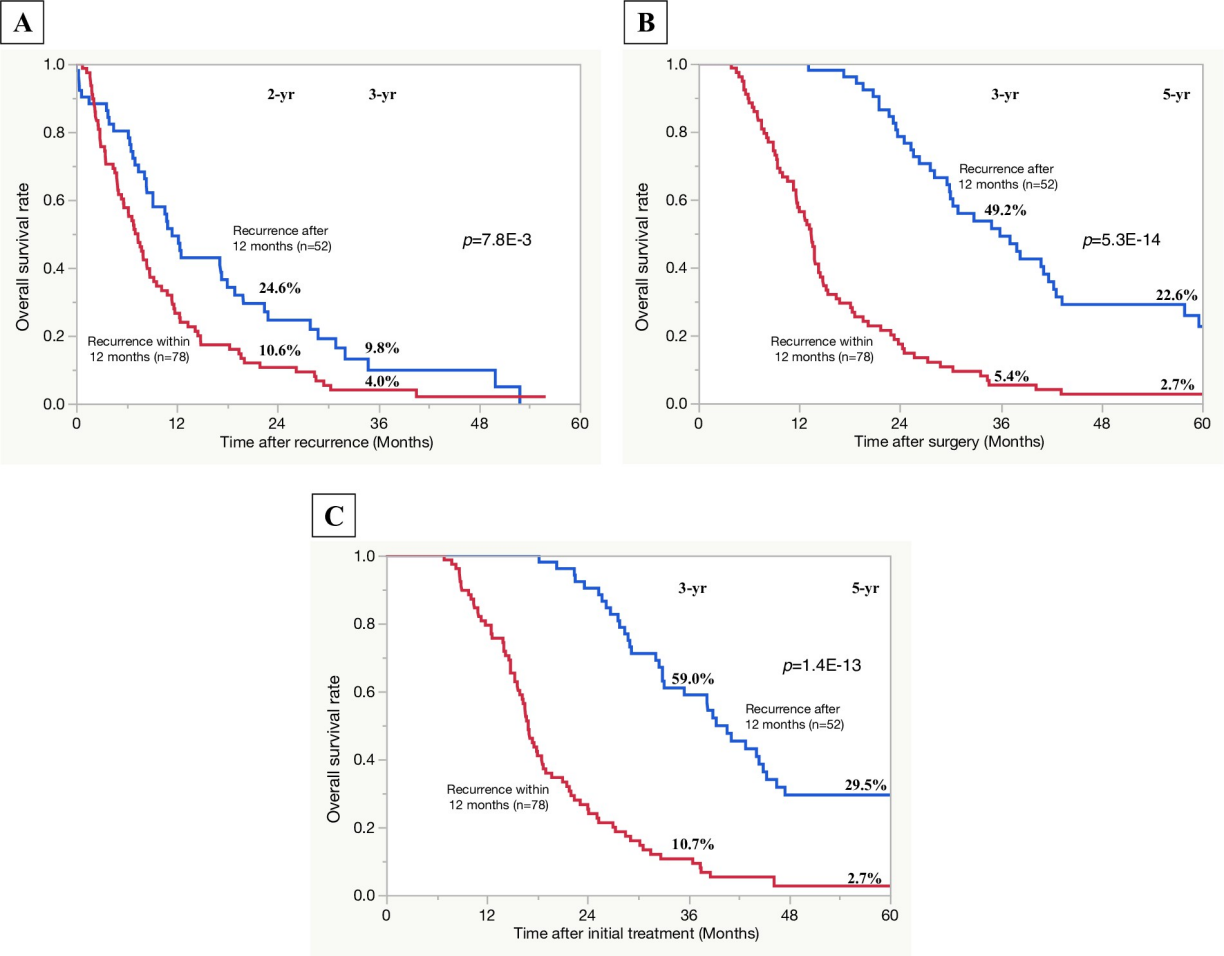
Comparison of survival curves based on the period of recurrence development from surgery (recurrence after 12 months vs. recurrence within 12 months). A: post-recurrence survival rates; B: overall survival rates after surgery; C: overall survival rates after initial treatment, *p* value was expressed in exponential notation.

**Fig 2:**
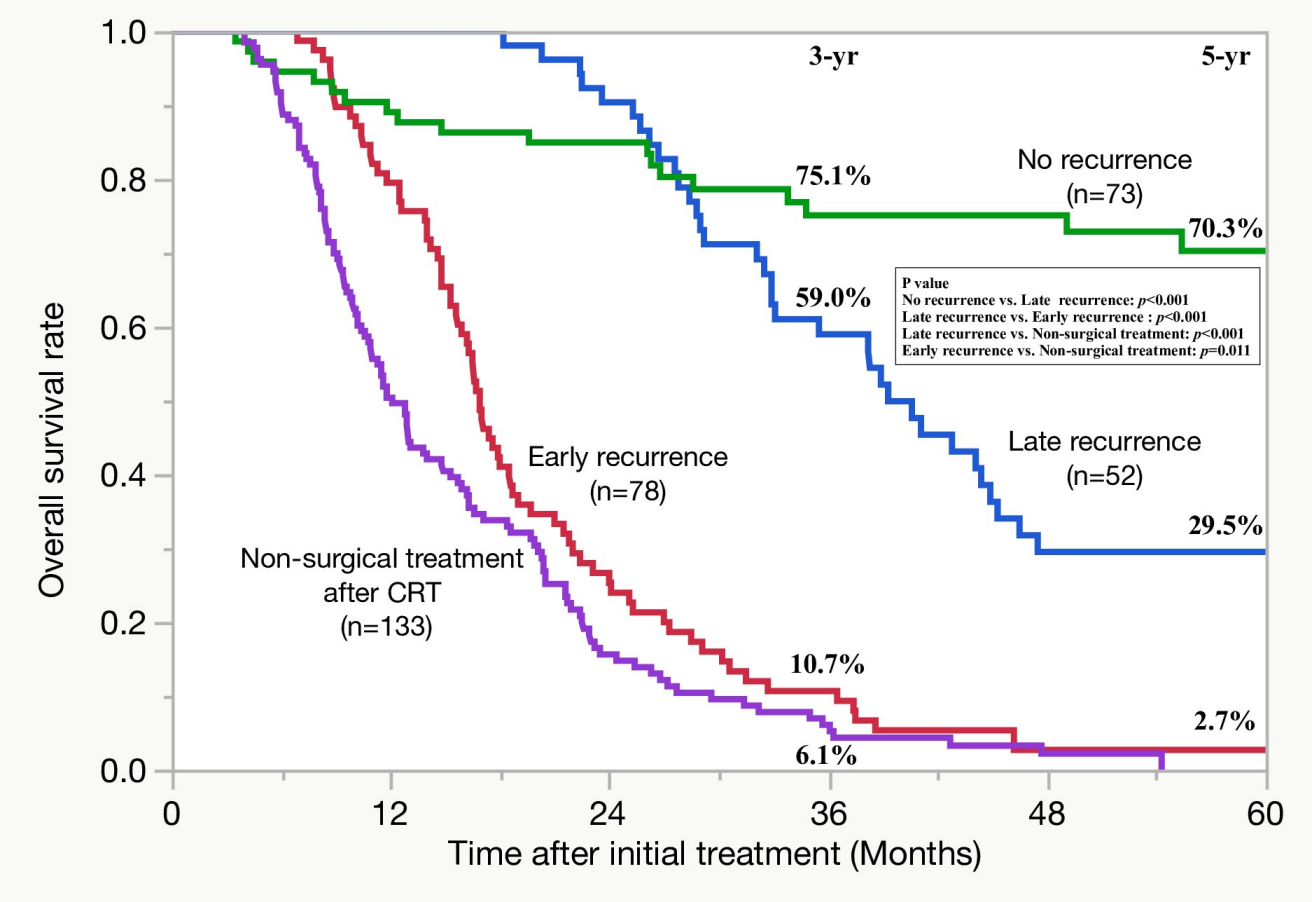
Comparison of the overall survival curves after initial treatment between the early and late and no recurrence and non-surgical treatment after CRT groups.

For 130 patients who developed tumor recurrence, the optimal cut-off threshold for differentiating early and late recurrence after surgery was 12 months (*p* = 7.8×10^−3^) when using the minimum *p*-value approach based on the difference of PRS (S1 Fig). Rates of PRS, OS after surgery, and that after initial treatment were significantly lower in the 78 patients who had an initial recurrence within 12 months than in the 52 patients who had an initial recurrence after more than 12 months (Fig 1). Therefore, 78 patients who had an initial recurrence within 12 months were defined as the early recurrence group, and 52 who had an initial recurrence after more than 12 months were defined as the late recurrence group. Fig 2 shows the comparison of OS rates after initial treatment between the early and late groups and no recurrence group (n = 73) and unresected cases after preoperative-CRT (non-surgical treatment after CRT group) (n = 133). OS rates after initial treatment in the late recurrence group were significantly higher than that in the non-surgical treatment after CRT group (3-, 5-year rates of OS: 59.0, 29.5 vs. 6.1, 0%, *p*<0.001). OS rates after initial treatment in the early recurrence group were significantly higher than that in the non-surgical treatment after groups (3-, 5-year rates of OS: 10.7, 2.7% vs. 6.1, 0%, *p* = 0.011), although there seemed to be not a marked difference in the 5-year OS rate between the two groups.

### Comparison of the recurrence form between the early and late recurrence

Table 1 shows a comparison of the initial recurrence form between the early and late recurrence groups. Liver metastasis was more prevalent in the early recurrence group than in the late recurrence group (early recurrence vs. late recurrence: 39.7 vs. 15.4%, *p* = 0.003). In contrast, lung metastasis was more prevalent in the late recurrence group than in the early recurrence group (early recurrence vs. late recurrence: 19.2 vs. 34.6%; *p* = 0.048).

**Table 1:** Comparison of the recurrence form between early recurrence and late recurrence.

	Early recurrence (n = 78)	Late recurrence (n = 52)	*p* value
Site of recurrence			
**Liver metastasis (yes/no)**	**31 (39.7%)/47**	**8 (15.4%)/44**	**0.003**
Peritoneal dissemination (yes/no)	20 (25.6%)/58	10 (19.2%)/42	0.398
**Lung metastasis (yes/no)**	**15 (19.2%)/63**	**18 (34.6%)/34**	**0.048**
Local recurrence (yes/no)	10 (12.8%)/68	13 (25.0%)/39	0.078
Lymph nodal metastasis (yes/no)	3 (3.9%)/75	4 (7.7%)/48	0.437

### Univariate analysis of predictive risk factors for early recurrence

To identify perioperative risk factors for early recurrence in the patients indicated for surgery, patients’ characteristics and clinical response of preoperative CRT were compared between the early recurrence group (n = 78) and the others (n = 125). In terms of patient characteristics before treatment, no significant difference was demonstrated by comparing age, sex, performance status (PS), tumor location, degree of tumor extension, status of lymph nodal metastasis, clinical stage, and chemotherapeutic agents of CRT. In terms of tumor resectability, there was not significant difference in the resectability before CRT and that before surgery between the two groups. Among the 203 patients with resection after CRT, 13 (6.4%) showed tumor down-staging (e.g., from UR-LA to BR) after CRT and 15 (7.4%) did tumor up-staging (e.g., from BR to UR-LA) after CRT. There was also no significant difference in the frequency of tumor down-staging and that of up-staging between the two groups. Among the patient conditional factors, NLR and PNI before and after CRT were not significantly different between the two groups. In terms of clinical response to preoperative CRT, serum CA19-9 level before surgery were significantly higher in the early recurrence group than in the others (early recurrence vs. others: 59.2 and 26.5 U/ml, *p* = 0.004), although there was no significant difference in the serum CA19-9 level before CRT (early recurrence vs. others: 187.6 and 125.4 U/ml, *p* = 0.128) (Table 2). There was not significant difference in the tumor size before CRT and surgery and reduction rate in tumor size during preoperative treatment.

**Table 2:** Patient characteristics and clinical response of preoperative chemoradiotherapy in the localized pancreatic cancer patients with curative-intent resection according to the occurrence of early recurrence after surgery.

	Early recurrence	Others	*p* value
	(n = 78)	(n = 125)	
**Patient characteristics before treatment**			
Age	67 (40–86)	67 (41–85)	0.659
Sex (Male/Female)	40/38	76/49	0.183
Performance status (0/1/2/3)	45/28/4/1	86/36/3/0	0.203
Tumor location (Ph/Pb/Pt)	52/11/15	98/12/15	0.183
Degree of tumor extension (T1/T2/T3/T4)	1/1/33/43	1/2/70/52	0.283
Lymph nodal metastasis (N0/N1a, N1b)	59/19	104/21	0.192
cStage before CRT (IA/IB/IIA/IIB/III)	1/1/29/4/43	0/1/59/13/52	0.152
Resectability before CRT (R/BR-PV/BR-A/UR-LA)	23/9/13/33	45/23/21/36	0.201
Resectability before surgery (R/BR-PV/BR-A/UR-LA)	20/11/13/34	49/16/22/38	0.165
Tumor down-staging (yes/no)	6 (7.7%)/72	7 (5.6%)/118	0.568
Tumor up-staging (yes/no)	8 (10.3%)/70	7 (5.6%)/118	0.272
Chemotherapeutic agents of CRT (G-CRT/GS-CRT)	28/50	36/89	0.292
**Patient conditional factor during CRT**			
NLR before CRT	2.5 (1–13.7)	2.4 (0.7–5.8)	0.118
NLR after CRT	3.2 (1.1–14.8)	3.2 (0.6–13.7)	0.477
PNI before CRT	47.8 (35.7–58.8)	46.7 (35.4–60)	0.956
PNI after CRT	44.3 (25.5–53.5)	44.7 (22.7–54.7)	0.472
**Clinical response of CRT**			
Interval from initial treatment to surgery (Months)	3.5 (2.3–11.5)	3.7 (1.5–17.2)	0.529
Serum CA19-9 level before CRT (U/ml)	187.6 (0.1–9127)	125.4 (0.2–17268.9)	0.128
**Serum CA19-9 level before surgery (U/ml)**	**59.2 (0.7–688.5)**	**26.5 (0.1–2406.3)**	**0.004**
Reduction rate in serum CA19-9 level during preoperative treatment (%)	68.0 (-1987.0–99.2)	76.8 (-183.2–99.8)	0.512
Tumor size* before CRT (mm)	57.5 (26.8–137.6)	54.7 (13.2–125.3)	0.078
Tumor size* before surgery (mm)	46.8 (19.1–107.8)	43.6 (19.7–104.7)	0.051
Reduction rate in tumor size during preoperative treatment (%)	13.1 (-58.6–61.2)	13.4 (-124.4–70.9)	0.928

CRT; chemoradiotherapy, R; resectable, BR; borderline resectable, UR-LA; locally advanced unresectable, G-CRT; gemcitabine-based CRT, GS-CRT; gemcitabine plus S-1 based CRT, NLR; neutrophil-to-lymphocyte ratio, PNI: prognostic nutritional index,

*Tumor size: sum of long and short axis of tumor

In terms of surgical outcomes, total blood loss during surgery was significantly greater in the early recurrence group than in the others (early recurrence vs. others: 1043 vs. 823 ml; *p* = 0.044). The frequency of administering the planned adjuvant chemotherapy was not significantly different between the two groups (early recurrence vs. others: 82.1% vs. 85.6%; *p* = 0.502).

In the comparison of histopathological factors, the degree of tumor extension was significantly higher in the early recurrence group than in the others (the rate of pT3-T4 in early recurrence vs. others: 87.2 vs. 60.8%; *p*<0.001) (Table 3). The degree of venous invasion and that of neural invasion were significantly higher in the early recurrence group than in the others (rate of v1-3 and that of ne1-3 in early recurrence vs. others: 35.9 and 78.2% vs. 21.6 and 59.2%; *p* = 0.027 and *p* = 0.005, respectively). As for the histological response to preoperative CRT, the frequency of grade 3 or more histological response, indicating pathological CR (pCR) or near-pCR, was significantly lower in the early recurrence group than in the others (the rate of grade 3 or more in early recurrence vs. others: 14.1 vs. 29.6%; *p* = 0.009).

**Table 3:** Surgical outcomes and histopathological characteristics in the localized pancreatic cancer patients with curative-intent resection after preoperative CRT according to the occurrence of early recurrence after surgery.

	Early recurrence	Others	*p* value
	(n = 78)	(n = 125)	
**Surgical outcomes**			
Operative procedures (PD/DP/TP)	61/17/0	105/19/1	0.314
Combined resection of SMV/PV (yes/no)	64/14	98/27	0.526
Combined reaction of hepatic artery* (yes/no)	5/73	14/111	0.326
Combined resection of celiac trunk (yes/no)	3/75	5/120	>0.999
Operative duration (min)	548 (203–842)	536 (195–782)	0.683
**Blood loss (ml)**	**1043 (106–11937)**	**823 (60–5830)**	**0.044**
Postoperative complications** (yes/no)	17/61	36/89	0.265
**Adjuvant chemotherapy**			
Performed	64 (82.1%)	107 (85.6%)	0.502
Not performed	14 (17.9%)	18 (14.4%)	
Chemotherapeutic agents of adjuvant chemotherapy (G/S1/GS)	28/34/2	51/55/1	0.537
**Histopathological characteristics**			
**Degree of tumor extension (pT0-T2/T3-T4)**	**10/68**	**49/76**	**<0.001**
Lymph nodal metastasis (pN0/N1a, N1b)	50/28	95/30	0.070
Degree of lymphatic invasion (ly0/ly1-3)	47/31	83/42	0.376
**Degree of venous invasion (v0/v1-3)**	**50/28**	**98/27**	**0.027**
**Degree of neural invasion (ne0/ne1-3)**	**17/61**	**51/74**	**0.005**
Surgical margin (R0/R1)	63/15	109/16	0.220
**Histological response to preoperative treatment (Grade 3, 4 /Grade 1, 2)**	**11/67**	**37/88**	**0.009**

PD; pancreaticoduodenectomy, DP; distal pancreatectomy, TP; total pancreatectomy, SMV; superior mesenteric vein, PV; portal vein, * hepatic artery; common hepatic artery and/or proper hepatic artery

** Postoperative complications; Clavian-Dindo classification grade IIIa or more,

### Multivariate regression analysis of predictive risk factors for early recurrence

**Fig 3:**
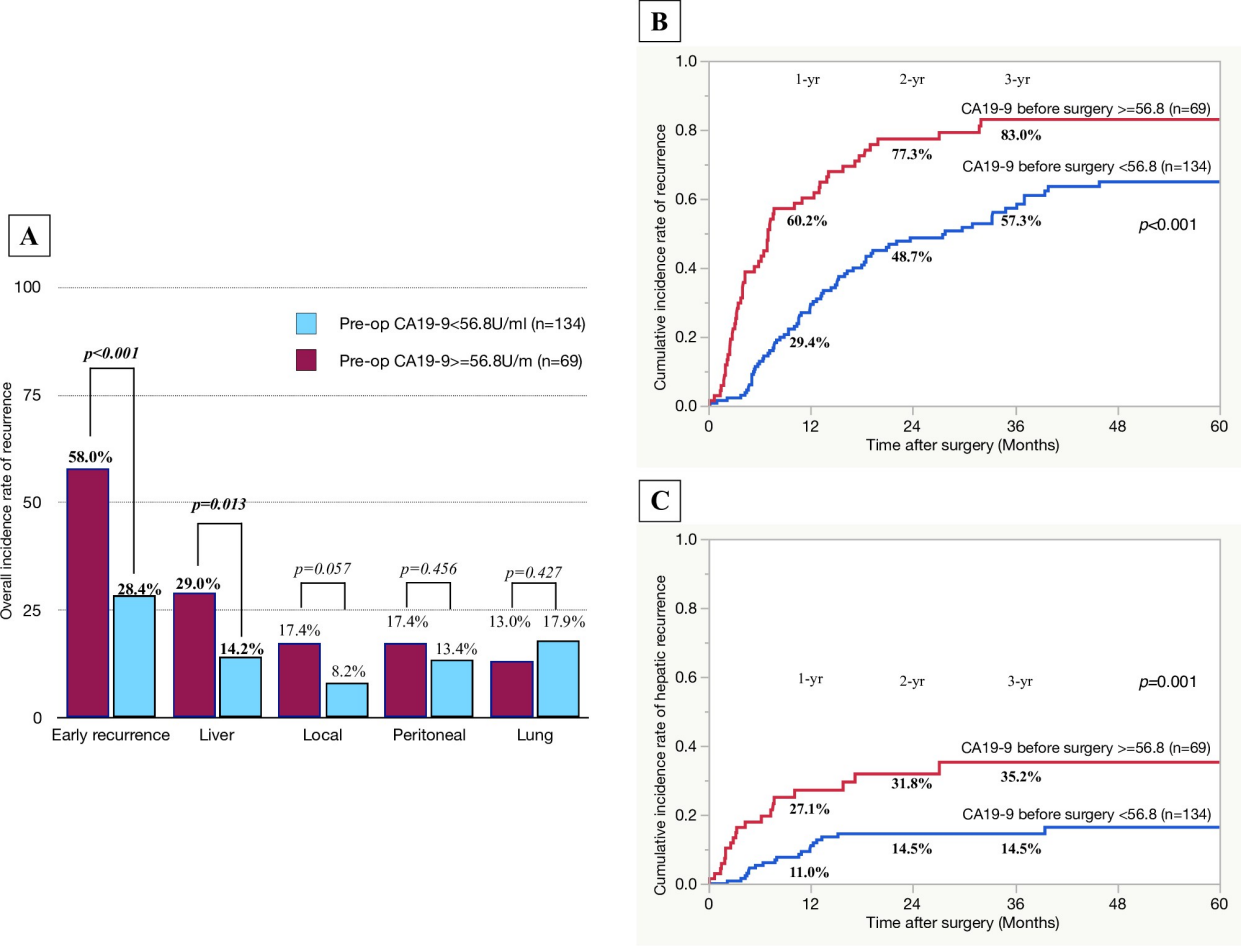
Comparison of overall and cumulative incidence rate of recurrence after surgery based on the serum level of CA19-9 level before surgery (= >56.8/<56.8 U/ml). A: overall incidence rate of early recurrence and site of recurrence after surgery; B: cumulative incidence rate of recurrence after surgery; C: cumulative incidence rate of hepatic recurrence after surgery.

**Fig 4:**
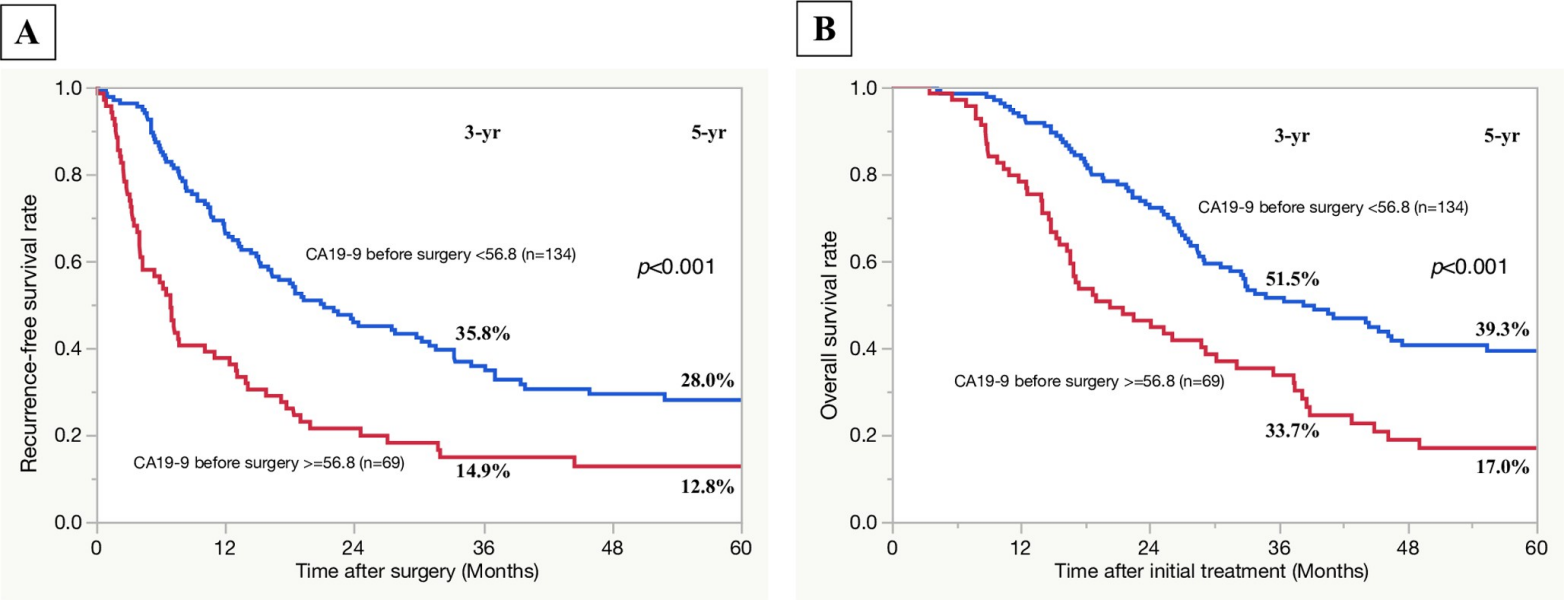
Comparison of recurrence-free and overall survival curves based on the serum level of CA19-9 level before surgery (CA19-9 before surgery = >56.8 vs. <56.8 U/ml). A: recurrence-free survival rates; B: overall survival rates after initial treatment.

For serum CA19-9 level before surgery, the area under the ROC curve (AUC) was 0.620 and the best cut-off value for predicting early recurrence was 56.8 U/ml with a sensitivity of 51.3% and specificity of 76.8%. The best cut-off value for intraoperative blood loss (AUC = 0.584) was 1300 ml with a sensitivity of 43.6% and specificity of 75.2%. Variables, of which the *p*-values were less than 0.05 by univariate analysis, were included as covariates in a multivariate regression analysis (Table 4). In a multivariate analysis, serum level of CA19-9 before surgery > = 56.8 U/ml (OR: 3.07, 95%CI: 1.65–5.73, *p*<0.001) was an independent risk factor associated with early recurrence after surgery. To validate the usefulness of the significant predictive factor, the overall incidence rate of early recurrence and site of initial recurrence were compared according to the presence or absence of the factor. The serum CA19-9 level before surgery > = 56.8 U/mL (n = 69) was associated with significantly higher overall incidence rate of early recurrence after surgery and that of hepatic recurrence (serum CA19-9 level before surgery > = 56.8 U/mL vs. <56.8 U/mL: 58.0 and 29.0% vs. 28.4 and 14.2%; *p*<0.001 and *p* = 0.013, respectively) (Fig 3A). The overall incidence rate of local recurrence after surgery was higher in the patients with serum CA19-9 level before surgery > = 56.8 U/mL than in those with serum CA19-9 level before surgery <56.8 U/m, but the difference was not statistically significant. The overall incidence rates of peritoneal and pulmonary recurrence were comparable between the two groups. The 1-and 2-year cumulative incidence rates of recurrence and those of hepatic recurrence were significantly higher in the patients with serum CA19-9 level before surgery > = 56.8 U/mL than in those with serum CA19-9 level before surgery <56.8 U/mL (Fig 3B and 3C). Serum CA19-9 level before surgery > = 56.8 U/mL was associated with significantly lower rates of recurrence-free and overall survival (Fig 4).

**Table 4:** Multivariate analysis of perioperative factors contributing to early recurrence after surgery in the localized pancreatic cancer patients with curative-intent resection after preoperative CRT.

Predictor	OR	95%CI	*p* value
**Serum CA19-9 level before surgery (U/ml) (> = 56.8/<56.8)**	**3.07**	**1.65–5.73**	**<0.001**
Blood loss (ml) (> = 1300/<1300)	1.86	0.98–3.50	0.057

## Discussion

The current study explored the optimal cut-off threshold to differentiate early and late recurrence and to identify predictive factors for early recurrence in 203 patients who underwent curative-intent resection after preoperative CRT. We confirmed that a recurrence-free interval of 12 months after surgery was the optimal threshold for differentiating between early and late recurrence after surgery. Patients with early recurrence had significantly higher prevalence of hepatic recurrence and lower post-recurrence and overall survival rates than the late recurrence group. These results indicate that early recurrence within 12 months is an important condition with a dismal prognosis in patients who underwent curative-intent resection after preoperative CRT. Because there was no significant difference in the proportion of initial tumor resectability before preoperative CRT and surgery between early and late recurrence, we speculate that this selected population of patients had tumors with potential aggressive behavior and concurrent occult metastasis which developed rapid progression after surgery.

Various cut-off values have been used to categorize patients with PDAC who underwent up-front surgery on timing of recurrence, for instance, 6 months by Sugiura et al. [18] and Matsumoto et al. [6], 8 months by Niedergethmann et al. [19], and 12 months by Nishio et al. [20] and Zhai et al. [21]. However, these previous studies were not performed based on the statistical assessment of the best cut-off value to differentiate prognosis. Two previous studies by Yamamoto et al. [8] and Groot et al. [7] revealed that the 12-month period after surgery was the optimal threshold for differentiating between early and late recurrence using the minimum *p*-value approach in patients with initially resectable PDAC who underwent up-front surgery. Yamamoto et al. established an optimal cut-off for differentiating early and late recurrence based on OS after surgery [8], while Groot et al. used the difference in post-recurrence survival to define early and late recurrence to avoid the potential bias of OS being better in the late recurrence group as this group already has a long recurrence-free survival [7]. In the present study, therefore, we used the difference in post-recurrence survival to define an optimal threshold with the minimum *p*-value approach as Groot. et al. proposed [7], and adopted a recurrence-free interval of 12 months after surgery as an optimal cut-off for differentiating early and late recurrence in the patients who underwent curative-intent resection after preoperative CRT.

Little is known about the clinicopathological features and predictive factors of early recurrence in patients with PDAC undergoing curative-intent resection after preoperative CRT. A multivariate regression analysis showed serum level of CA19-9 before surgery (≥56.8 U/ml) was an independent preoperative risk factor for early recurrence after surgery in the present study. Preoperative CA19-9 level has been reported to be an independent predictive factor for early recurrence of resectable PDAC after up-front surgery [8, 18, 20]. Yamamoto et al.[8] and Sugiura et al.[18] reported that a preoperative CA19-9 value ≥100 U/ml was found to be a significant predictor among preoperative parameters for early recurrence and poor prognosis after up-front surgery for PDAC [8, 18]. Groot et al.[7] demonstrated that preoperative CA19-9 >210 U/mL, Charlson age-comorbidity index > = 4, tumor size >3.0cm on computed tomography were independent predictors for early recurrence within 12 months after surgery in a total of 975 patients with primary resectable PDAC who underwent pancreatectomy. Our results clearly demonstrates that the preoperative CA19-9 value is an independent predictive factor for early recurrence in a total of 203 patients with PDAC undergoing curative-intent resection after preoperative CRT and can become a useful parameter for decision-making regarding surgical indication after preoperative CRT. Aoki et al. reported that decreased CA19-9 levels after neoadjuvant therapy for the patients with PDAC predicted a better prognosis, with a low incidence of hepatic recurrence after surgery, which supports our results [22]. Because we demonstrated that hepatic recurrence was more prevalent in the early recurrence group than in the late recurrence group, elevated levels of serum CA19-9, even after preoperative CRT, may reflect systematic expansion and the existence of micrometastases in distant lesions, particularly hepatic lesions [23].

In recent years, patient conditional factors have become important when making decisions of surgical resection because they are associated with intolerance to the preoperative CRT and poor overall prognosis. We previously reported that a poor PS before preoperative CRT and a low value of PNI after preoperative CRT were associated with worsened prognosis in patients with PDAC who received preoperative CRT [24, 25]. In the present study, however, the PS before preoperative CRT and the PNI after preoperative CRT were not significantly different between the early recurrence group and the others, suggesting that the patient conditional factors were not closely associated with early recurrence in PDAC patients with resection after preoperative CRT. Among patient conditional factors, serum albumin reflects the nutritional status of patients with PDAC, and a low level of preoperative serum albumin has been reported to be an independent poor prognostic factor after resection for patients with PDAC [26]. Interestingly, Alessandro et al. demonstrated that increased preoperative CA19-9 levels (CA19-9 > = 32 U/ml) were significantly associated with pathological lymph node metastasis, and this ability was lost in the presence of hypoalbuminemia, suggesting that level of serum albumin affect the accuracy of preoperative CA19-9 levels in predicting oncological outcomes for patients with PDAC [27]. In the present study, however, the levels of serum albumin before and after preoperative CRT were not significantly different between the early recurrence group and the others (early recurrence vs. others before and after preoperative CRT: 4.0 and 3.9 vs. 3.9 g/dL and 3.9 g/dL; *p* = 0.384 and *p* = 0.977, respectively), and the serum CA19-9 level before surgery > = 56.8 U/mL was associated with significantly higher rate of early recurrence after surgery even in the presence of hypoalbuminemia. Our results indicate that serum level of CA19-9 before surgery was a stronger factor in predicting early recurrence after surgery than the patient nutritional status after preoperative CRT.

In the present study, we demonstrated that a greater amount of intraoperative blood loss was significantly associated with early recurrence after preoperative CRT followed by surgery. It has been reported that large intraoperative blood loss was an independent prognostic factor for tumor recurrence and death for major malignant tumors such as hepatocellular carcinoma [28], but its prognostic significance in patients with PDAC remains controversial because of the lack of evidence [29, 30]. It was reported that large intraoperative blood loss increased the risk of postoperative morbidity in patients with PDAC who underwent pancreatectomy [31]. Therefore, an increased morbidity rate is potentially associated with a delay in the initiation or decreased frequency of adjuvant chemotherapy, which is known to be associated with poor prognosis in PDAC patients with surgery [32]. In our study, however, the morbidity rate and frequency of conducting the planned adjuvant chemotherapy were comparable between the early recurrence group and the others. It was reported that colorectal cancer patients who received blood transfusions for excessive intraoperative blood loss showed exaggerated postoperative systemic induction of interleukin-6, triggering tumor growth factors and resulting in a poor long-term prognosis [33]. We speculate that the excess intraoperative blood loss reflected the invasiveness of the surgical procedure and the intense surgical stress, which are correlated with possible causes of adverse effects such as systemic induction of inflammatory cytokines and inflammation-induced anti-tumor immunosuppression in the early recurrence group.

In the comparison of histopathological factors, the frequency of grade 3 or more histological response, indicating pathological CR (pCR) or near-pCR, was significantly lower in the early recurrence group than in the others. Furthermore, the degree of tumor extension was significantly higher in the early recurrence group than in the others, although the degree of initial tumor extension before CRT was not significantly different between the two groups. These results indicate that the early recurrence is closely associated with the insufficient pathological tumor downstaging due to limited histological response of preoperative CRT. We previously reported that pCR or near-pCR contributed to achieving a high rate of R0 resection and improving recurrence-free and overall survival in patients with localized PDAC who underwent curative-intent resection after preoperative CRT [34]. The present study indicates that the pCR or near-pCR to preoperative CRT was an important pathological factor which is associated with significantly lower incidence of early recurrence. Further enhancement of the histological response to CRT is necessary for reducing the incidence of early recurrence and further improvement in the outcomes after preoperative CRT followed by surgery. It has been recently demonstrated that the systemic chemotherapy using FOLFIRINOX regimens followed by preoperative CRT, referred as total neoadjuvant therapy (TNT), addresses both occult metastases and positive margin risks and thus improves outcomes of patients with localized PDAC. Because extended duration of systemic chemotherapy using FOLFIRINOX before preoperative CRT has been reported to be closely associated with normalization of post-chemotherapy CA19-9 and major pathological response and thus predict postoperative survival [35], the TNT using FOLFIRINOX regimens might be a potential optimal treatment strategy which can reduce the incidence of early recurrence for patients with localized PDAC.

The main limitation of the present study is its retrospective nature and single-institution design. As for the minor limitation, there might be potential confounding factors after surgery such the use of non-contemporary chemotherapy after recurrence. Future multi-institutional studies are necessary to clarify the usefulness of CA19-9 before surgery and seek more potent factors for predicting the early recurrence after preoperative CRT followed by surgery.

## Conclusion

The present study demonstrates that an interval of 12 months after surgery is the optimal cut-off threshold between early and late recurrence in patients with PDAC who underwent curative-intent resection after preoperative CRT. Early recurrence was pathologically related to the presence of venous invasion and neural invasion and the limited histological response to preoperative CRT. A greater amount of intraoperative blood loss was significantly associated with early recurrence after surgery. Serum level of CA19-9 before surgery > = 56.8 U/ml was a strong independent predictive factor for early recurrence and associated with a significantly higher cumulative incidence rate of hepatic recurrence and lower rates of overall and recurrence-free survival. Our results indicate herein that careful observation and management by monitoring serum CA19-9 levels is necessary for the selection of surgical candidates after preoperative CRT, and high-quality surgical performance and minimization of intraoperative blood loss are crucial to prevent early recurrence after surgery.

## Supporting information

S1 Fig Minimum *p* value analysis demonstrating optimal cut-off threshold in every 3 months between early and late recurrence after surgery based on the difference of post-recurrence survival. (TIF)

S1 Dataset (XLSX)
