# Supplementary figures and images for: Predictive risk factors for early recurrence in patients with localized pancreatic ductal adenocarcinoma who underwent curative-intent resection after preoperative chemoradiotherapy
Source: PLoS One. 2022 Apr 4;17(4):e0264573. doi: 10.1371/journal.pone.0264573 (PMC8979444; doi:10.1371/journal.pone.0264573)

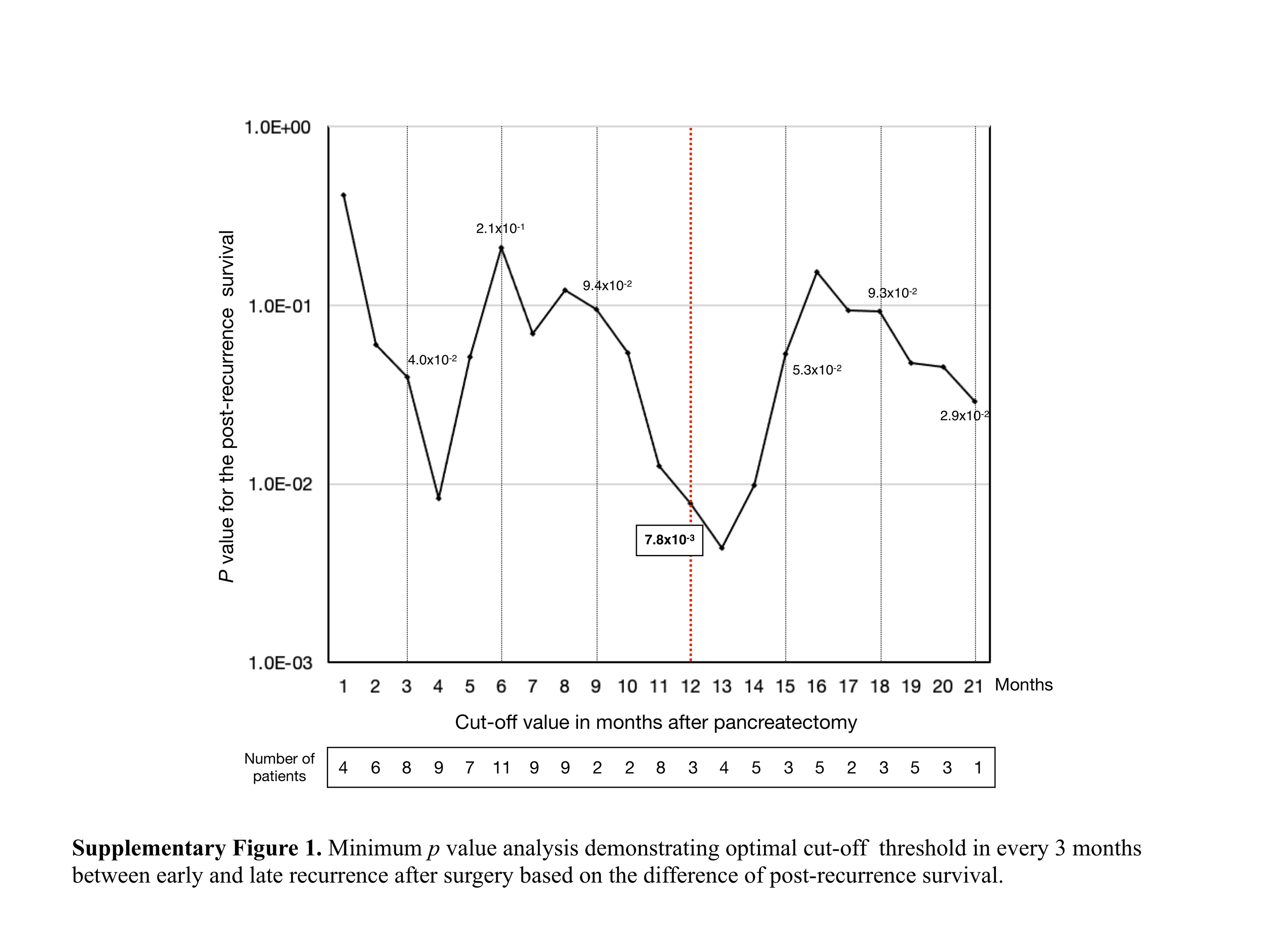

Supplement: S1 Fig — (TIF) [file pone.0264573.s001.tif]
